# LRRK2 and neuroinflammation: partners in crime in Parkinson’s disease?

**DOI:** 10.1186/1742-2094-11-52

**Published:** 2014-03-21

**Authors:** Isabella Russo, Luigi Bubacco, Elisa Greggio

**Affiliations:** 1 Department of Biology, University of Padova, Via Ugo Bassi 58/B, 35121 Pad ova, Italy

**Keywords:** LRRK2, Neuroinflammation, Microglia, Neurodegeneration, Parkinson’s disease, Dopaminergic neurons

## Abstract

It is now well established that chronic inflammation is a prominent feature of several neurodegenerative disorders including Parkinson’s disease (PD). Growing evidence indicates that neuroinflammation can contribute greatly to dopaminergic neuron degeneration and progression of the disease. Recent literature highlights that leucine-rich repeat kinase 2 (LRRK2), a kinase mutated in both autosomal-dominantly inherited and sporadic PD cases, modulates inflammation in response to different pathological stimuli. In this review, we outline the state of the art of LRRK2 functions in microglia cells and in neuroinflammation. Furthermore, we discuss the potential role of LRRK2 in cytoskeleton remodeling and vesicle trafficking in microglia cells under physiological and pathological conditions. We also hypothesize that LRRK2 mutations might sensitize microglia cells toward a pro-inflammatory state, which in turn results in exacerbated inflammation with consequent neurodegeneration.

## Introduction

Leucine-rich repeat kinase 2 (LRRK2) is a large multidomain protein belonging to the family of mammalian ROCO (Ras Of COmplex) proteins, and characterized by the presence of an enzymatic core, comprising ROC/GTPase, COR (C-terminus of ROC) and serine threonine kinase domains, and by multiple protein-protein interaction domains including ankyrin and leucine-rich repeat motifs at the N-terminus, and WD40 repeats at the C-terminus [1,2]. Although the physiological functions of LRRK2 are still unclear, the presence of both a GTPase and a kinase domain suggest a role in intracellular signaling, and the existence of different protein binding domains points to an additional function as a scaffolding protein for the assembly of protein complexes [3].

Mutations in LRRK2 cause autosomal-dominantly inherited Parkinson’s Disease, while more common variations can also act as risk factors for disease, accounting for 13% of all familial PD cases, and 1 to 2% of all sporadic PD cases [1,4,5]. Despite intensive research efforts, little is known about the pathogenic mechanism(s) of mutant LRRK2. Interestingly, LRRK2-associated PD is clinically and pathologically similar to sporadic PD, thus indicating overlapping pathways in both familial and sporadic PD [1]. Several mutations in LRRK2 clearly segregate with the disease, and, importantly, these mutations cluster within the two catalytic domains, suggesting that a change in enzymatic functions (GTPase and/or kinase) may mediate the pathogenic effects of LRRK2 [6]. R1441G/C/H mutations map to the ROC domain [4,7,8], Y1669C to the COR domain [1], and I2020T and G2019S mutations to the kinase domain [9,10]. In this frame, the G2019S mutation is by far the most common pathogenic LRRK2 mutation, and is responsible for more than 10% of familial PD cases and 1 to 2% of sporadic PD cases [11]. Several studies have shown that of all the LRRK2 mutations, only the G2019S mutation consistently increases kinase activity [12-14]. Specifically, *in vitro* phosphorylation assays from independent studies showed an approximately three fold increase in kinase activity in comparison with wild-type protein, whereas ROC-COR mutations display inconsistent effects on kinase, but have significantly lower GTPase activity [15-18]. Conversely, the effect of the I2020T mutation on kinase activity is less clear, with studies observing both increased [19] and decreased [20,21] activity. However, the effects of LRRK2 mutations on kinase activity have been mainly examined *in vitro* by monitoring autophosphorylation or phosphorylation of model peptides, making it difficult to understand the real effects of mutations on LRRK2 physiological functions. Interestingly, a recent paper by Sheng and colleagues reported that autophosphorylation on serine 1292 is a direct indicator of LRRK2 kinase activity *in vivo*, and that additional mutations, other than G2019S, appear to increase autophosphorylation at this site [22], consistent with an *in vitro* study focused on autophosphorylation [23]. Monitoring phosphorylation of serine 1292 in cells represents an important tool for future studies addressing pathways and signaling networks that relate to LRRK2 kinase function.

In the brain, LRRK2, both mRNA and protein, has been detected in specific regions including the cortex, striatum, hippocampus, and with substantial lower expression, in the substantia nigra pars compacta (SNpc) [24-26]. LRRK2 has been reported to localize within a wide range of vesicular and membranous structures, such as mitochondria, endoplasmic reticulum, and Golgi apparatus, as well as the endolysosomal system and synaptic vesicles [25,27-29]. Although LRRK2 protein is expressed in various brain cell types such as neurons, microglia, and astrocytes [30], the vast majority of studies to date have focused on investigating LRRK2 functions in neurons, being the relevant cell type that degenerates in PD. The physiological functions of LRRK2 in neuronal cells have been linked to vesicular trafficking [31,32], cytoskeletal dynamics [33,34], mitochondrial function [35,36], apoptosis [37], and regulation of the autophagy pathway [38-41]; however, how LRRK2 mutations cause neurodegeneration in PD is currently under debate.

### Effects of reactive microglia and neuroinflammation in the pathogenesis of PD

It is now well established that chronic inflammation is a prominent feature of several neurodegenerative disorders, including PD [42,43]. The inflammatory response is driven by the activation of resident macrophages, local invasion of circulating immune cells, and production of cytokines, chemokines, and reactive oxygen species, all essential to recruit cells of the immune system to the compromised area [42]. Microglia cells, the resident macrophages of the brain, constitute the first barrier of the innate immune response in the brain, and are considered to be key players during neuroinflammation [42,44,45]. Microglia cells continually survey the microenvironment, and upon detection of abnormal changes in the surrounding tissue, they rapidly become activated, secrete inflammatory mediators, and migrate to the damaged area to eliminate pathogens or to phagocyte dead cells and/or aggregated proteins [46,47]. Although a well-regulated inflammatory process is essential for tissue repair and CNS integrity, an excessive and protracted inflammatory response can turn cytotoxic, leading to significant tissue and cellular damage, thus promoting the progression of the disease [42,48].

It has been widely reported that chronic inflammation can contribute to the degeneration of dopaminergic neurons and to the progression of PD [48,49]. Elevated levels of pro-inflammatory cytokines (tumour necrosis factor (TNF)-α, interferon (IFN)-γ, interleukin (IL)-1β, and IL-6) have been found in the cerebrospinal fluid, striatum and SNpc of experimental animal models and of post-mortem brains from patients with PD [48-50]. Moreover, the presence of extensive proliferation of reactive and phagocytic macrophages positive for human leukocyte antigen (HLA) has been shown around dopaminergic neurons in the SNpc of patients with PD [51], in animal models, and in patients with parkinsonism after 1-methyl-4-phenyl-1,2,3,6-tetrahydropyridine (MPTP) exposure [52,53], further indicating that microglia cells and their mediators might lead to neuronal damage and degeneration [54,55].

Microglia-mediated dopaminergic neurodegeneration is supported by numerous studies. In fact, this class of neurons exhibits a reduced antioxidant capacity and enhanced sensitivity to pro-inflammatory mediators, probably as a result of the high density of microglia cells present in SNpc compared to other brain regions [56-59]. Ouchi and colleagues reported that microglia activation in the midbrain is positively correlated with dopaminergic neuronal death and motor symptom severity in the early stage of PD [60], supporting the notion that reactive microglia exacerbate the progression of the disease.

Supporting evidence for a direct role of inflammation in PD has recently emerged from genome-wide association studies (GWAS). Specifically, polymorphisms in genes encoding inflammatory cytokines such as *TNF-α* and *IL-1β*, and cell-surface human leukocyte antigen (*HLA*) are associated with a higher risk of developing PD [61-64]. These genes are predominantly expressed in glial cells, and are directly involved in the inflammatory process [65]. Although it is still controversial whether inflammation is causative or a secondary effect of earlier pathological events of PD, there is an increasing recognition of neuroinflammation as a major player in the pathology of this disease.

Remarkably, *LRRK2* has been identified by GWAS as one of the susceptibility genes for leprosy and possibly for Crohn’s disease [66-68], two illnesses with a strong inflammatory component. Based on these genetic findings, a number of recent studies have explored the putative role of LRRK2 in microglia cells and the impact of LRRK2 mutations in the inflammatory response associated with PD.

### What is the role of LRRK2 in microglia cells?

Microglia are highly dynamic cells with vastly branched and motile cell processes, which constantly screen the brain parenchyma, ready to detect any changes in the tissue [47]. In response to pathological stimuli such as lipopolysaccharide (LPS), aggregated proteins, or dead neurons, microglia cells switch from a resting and ramified phenotype into an ameboid, activated phenotype [47,69,70]. Once they have migrated toward a damaged area, they initiate a repairing program through the release of inflammatory mediators and the removal of debris by phagocytosis [71]. A number of LRRK2 pathways have been described in neuronal cells [72-74], but here we will discuss the potential roles of this protein in the physiological and pathological functions of microglia cells.

#### Immune-related cellular pathways

Recent findings indicate elevated LRRK2 mRNA and protein expression in immune cells, particularly in microglia and astrocytes from human and rodent brain [30,75,76], and in peripheral blood mononuclear cells, mainly in B cells, dendritic cells, and macrophages [77,78]. Giesert *et al*. investigated potential LRRK2 mRNA splice variants in brain cells [26]; they observed that both neurons and astrocytes (but not microglia) express a transcript variant containing the alternative exon 42a, which results in a premature stop codon and a predicted protein of 2152 amino acids long. This finding is intriguing, as it suggests that LRRK2 function(s) in microglia cells may be at least partly different from those exerted in other brain cell types.

Interestingly, Moehle and colleagues observed a robust induction of LRRK2 protein in microglia cells of mouse SNpc or striatum after LPS-induced inflammation [30]. Furthermore, *in vitro* studies reported increased LRRK2 protein expression in microglia cell cultures after an inflammatory stimulus induced by LPS or IFN-γ [79], but not after HIV-1 Tat protein-induced inflammation [76]. In addition to increased expression levels, LRRK2 immunoprecipitated from LPS-inflamed brains was more active in *in vitro* autophosphorylation assays [30], possibly indicating that the protein is switched into an active conformation. Although the exact mechanism is not clear, two different studies reported that HIV-1 Tat protein-induced inflammation of BV2 cells or Toll-like receptor (TLR) agonists (but not TNF-α treatment of RAW264.7 macrophages) induced marked phosphorylation of LRRK2 at serines 910 and 935 [76,80], a state that is possibly associated with an active LRRK2 form. Taken together, these data suggest that LRRK2 expression and enzymatic activity are required during an inflammatory response; however, the specific pathways and signaling cascades that LRRK2 orchestrates have not been characterized.

Additional studies further support a role for LRRK2 as regulator of inflammation. Murine microglia cells with inhibited or down-regulated LRRK2 showed attenuated levels of LPS-induced inflammatory mediators at the mRNA and protein level, including TNF-α, IL-1β, IL-6 and inducible nitric oxide synthase (iNOS) [30,81]. In particular, LRRK2 knockdown microglia exhibited a significant reduction of nuclear factor kappa B (NF-κB) transcriptional activity following TLR-mediated immune signaling induced by LPS, and an increase of DNA-binding activity of the NF-κB p50 inhibitor subunit [81]. As NF-κB activity is essential for induction of pro-inflammatory cytokines [82-84], LRRK2 might control its activation and the consequent transcription of pro-inflammatory mediators, through a yet undisclosed mechanism. In another study, Liu and colleagues demonstrated that LRRK2 can function as negative regulator of nuclear factor of activated T cells (NFAT) transcription factors, a class of molecules implicated in the inflammatory response in a large set of immune cells [85]. In particular, they observed that LRRK2 inhibits NFAT nuclear translocation in a TLR-independent manner, and thus inhibits the transcription of immune response genes via interaction with the NRON (noncoding (RNA) repressor of NFAT) complex in immune peripheral cells [62]. Although a significant amount of progress has been made, how LRRK2 modulates transcription factors and induction of inflammatory genes remains controversial and needs to be carefully explored.

**Figure 1:**
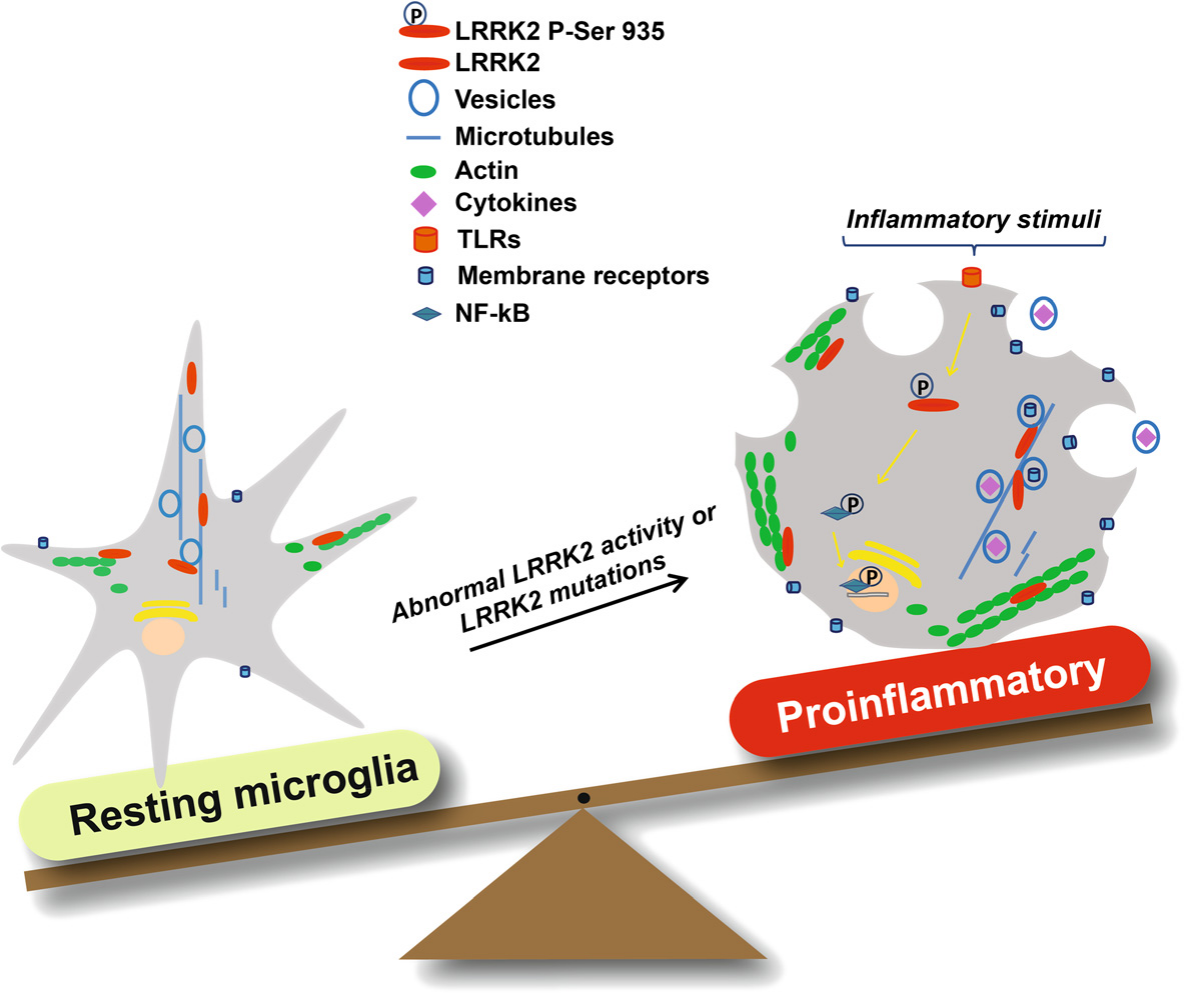
**Abnormal activity of or mutations LRRK2 might push microglia cells toward a pro-inflammatory phenotype.** Abnormal LRRK2 activity might modulate microglia cell activation and phagocytosis through hyperphosphorylation and hyperpolymerization of cytoskeleton components such as actin and β-tubulin. Moreover, LRRK2 might regulate the delivery of membrane receptors (CD11b and MHC- II) and inflammatory cytokines through regulation of transcription factors (such as NF-κB), and interaction and phoshorylation of vesicle-associated proteins such as Rab GTPase and NSF, thus driving microglia toward a reactive phenotype with enhanced cell activity and inflammation in response to inflammatory stimuli including LPS, environmental insults, and neuronal susceptibility.

Notably, Gillardon and colleagues [79] observed that LPS-activated microglia cells from LRRK2 R1441G knock-in mice exhibited increased expression and secretion of pro-inflammatory cytokines and reduced expression of anti-inflammatory cytokines compared with wild-type control microglia cells. Interestingly, gene expression profiling of LRRK2 R1441G microglia cells under unstimulated conditions revealed that several cytokines, chemokines, and membrane receptors regulating microglia activation were significantly increased compared with wild-type cells, indicative of a basal sensitization toward a pro-inflammatory phenotype. In addition, conditioned medium from LPS-stimulated LRRK2 R1441G microglia added to primary cortical neurons caused increased neuronal death compared with medium from wild-type LRRK2 microglia. Overall, these data indicate that LRRK2 is involved in the cellular pathways induced by inflammation, and that LRRK2 R1441G mutation might push microglia toward a pro-inflammatory state, which results, in turn, in exacerbated inflammation and consequent neurodegeneration in patients with PD (Figure 1).

Of note, Lopez de Maturana and colleagues recently reported the effects of pro-inflammatory LPS stimulus on epidermal cells from patients with PD who carried R1441G or G2019S LRRK2 mutations, and patients with idiopathic PD compared with controls [86]. Interestingly, these authors found that patient cells had a blunted response to LPS compared with control cells. However, whether immune cells respond to LPS priming in a similar manner remains to be determined.

#### Cytoskeleton reorganization

LRRK2 has been suggested to influence actin cytoskeleton and microtubule dynamics, and these functions might be implicated in the neuronal damage underlying PD [73,87-89]. Various studies have reported the involvement of LRRK2 kinase activity in neurite outgrowth and maintenance of neuronal processes. There seems to be general agreement that the LRRK2 G2019S mutation has an inhibitory effect on neurite outgrowth and branching [20,90-92]. In this context, Parisiadou and colleagues hypothesized that the G2019S mutation causes increased actin polymerization and ERM (ezrin, radixin, moesin) protein phosphorylation in neuronal filopodia, thus acting as a physical barrier that prevents the extensions of microtubule and leads, in turn, to inhibition of neuronal processes [73]. These observations find a link with the proposed function of LRRK2 in the context of the microtubule network. In particular, it was reported that LRRK2 interacts through its ROC/GTPase domain and phosphorylates neuronal β-tubulin both *in vitro* and *in vivo*, as demonstrated by reduced phosphorylation of β-tubulin immunoprecipitated from knockout compared with wild-type mouse brains [34,89,93]. Furthermore, *in vitro* LRRK2 enhances tubulin polymerization in the presence of microtubule-associated proteins [89,94], and phosphorylates tubulin-associated Tau [95], whereas *in vivo*, Tau is hyperphosphorylated in brains from R1441G trangenic mice [96]. Taken together, these data indicate that LRRK2 regulates the organization of the actin cytoskeleton and microtubule assembly.

Cytoskeleton reorganization is also a critical step for brain macrophage functions. Indeed, the rapid changes in cell morphology and cell activity that microglia cells undertake upon activation are governed by microtubules and by actin cytoskeleton remodeling [87,97-99]. Even microglia motility is a process that results from coordinated changes in the actin cytoskeleton and in the formation of cell-substrate adhesion sites. The actin cytoskeleton provides the driving force at the cell front, while the microtubule network regulates the rear retraction [97,99]. During migration, microglia cells exhibit long, thin extensions called filopodia, which are supported by tight and dynamic bundles of actin filaments that continuously polymerize and depolymerize to support the cell movement [97,100-104].

Supporting a role of LRRK2 in cytoskeleton reorganization during microglia activation, two recent studies reported that inflammatory stimuli failed to induce significant morphological changes of fine process extensions and cytoskeleton remodeling in LRRK2 knockdown or LRRK2 inhibitor-treated microglia cells [30,76]. Moreover, Moehle and colleagues observed *in vitro* that LRRK2 inhibition prevents microglial migration in response to ADP, a potent microglial chemoattractant [30]. Another study demonstrated that human LRRK2 G2019S and mouse LRRK2 knockout fibroblasts exhibit altered cell migration in culture, with LRRK2 G2019S fibroblasts migrating faster and LRRK2 knockout migrating slower compared with control cells [87]. As the G2019S mutation increases LRRK2 catalytic function, LRRK2 kinase activity appears to be required for cytoskeleton remodeling and for motility of microglia cells.

Microglia are the predominant phagocytes in the brain. They act as a surveillance system for microbes and pathological proteins, and remove apoptotic cells and cellular debris in the brain [70]. They engulf and digest microbes or pathological proteins by binding to them *via* specialized surface receptors (such as TLRs) and subsequently trapping them into a phagosome, which then fuses with a lysosome to be digested [46,105]. Phagocytosis induces dramatic changes in the shape and the movement of a cell, including extension of the membrane and actin filaments, through reorganization of the cytoskeleton [106]. Barcia and colleagues demonstrated using *in vivo* high-resolution imaging that the entire microglia cell body is involved in the formation of a phagocytic cup [107]. Moreover, imaging of fluorescent actin molecules during phagocytosis showed coordinated movement of the cytoskeleton in the cup, which is mediated by a combination of localized actin polymerization and depolymerization, together with the contraction of actin filament networks [108]. Interestingly, Marker *et al*. showed that LRRK2 might be involved in microglia phagocytosis. They found that pharmacological inhibition of LRRK2 attenuated microglial phagocytosis of fluorescent beads after an inflammatory stimulus. In addition, BV2-immortalized microglia cells co-cultured in the axonal compartment of primary neurons (to study phagocytosis of neuronal processes in the absence of cell bodies), and exposed to inflammatory conditions, completely phagocytosed and destroyed axonal arbors. By contrast, treatment of co-cultures with the LRRK2 inibitor IN-1 protected the axons and neuronal phagocytosis by clearing microglia [76].

Overall, these data suggest that LRRK2 regulates microglia activation, migration, and phagocytosis, although the molecular mechanisms underlying these processes are still unknown. One possibility is that LRRK2 orchestrates cytoskeletal components such as actin, tubulin. and ERM proteins, and that the pathological G2019S mutation causes hyperphosphorylation and hyperpolymerization of cytoskeleton components, which leads, in turn, to reactive microglia with enhanced cell activity, migration, and phagocytosis in response to pathological stimuli, thus contributing to the pathogenesis of PD (Figure 1).

#### Vesicle trafficking

Strong evidence supports a role for LRRK2 in the secretory and endocytic pathways. Multiple *in vivo* and *in vitro* studies have reported that LRRK2 overexpression or knockdown affects synaptic vesicle endocytosis and exocytosis [32,109,110]. In detail, LRRK2 modulates synaptic vesicle trafficking by interaction with pre-synaptic proteins such as actin, syntaxin, synaptic vesicle glycoprotein 2A (SV2A), *N*-ethylmaleimide-sensitive factor (NSF), and Synapsin I [31]; affects synaptic vesicles endocytosis through interaction with Rab5b and EndoA, critical components of the endocytic machinery [32,110]; and regulates the numbers of readily releasable vesicles and their release in hippocampal neurons by interaction and phosphorylation of Snapin protein [111]. Importantly, the G2019S and R1441G mutations were shown to increase the levels of dopamine receptor D1 on the membrane surface [112], supporting the notion that pathological LRRK2 activity might affect vesicle trafficking and release.

LRRK2 has also been reported to regulate the endocytic pathway involved in the autophagic-lysosomal process, an important clearance route for several aggregated or unfolded proteins. Interestingly, the LRRK2 *Drosophila* homolog interacts with Rab7, modulating the degradation of autophagosomes [27]. Human LRRK2 forms a complex with Rab7L1, an important component of the autophagic-lysosomal pathway [113]. In addition, LRRK2 interacting with Rab5 might also affect the early step of autophagosome formation [114].

Vesicle trafficking in secretory and endocytic pathways are well described in microglia cells during cytokine release, delivery of membrane receptors to the cell membrane, and phagocytosis [115,116]. Transport of inflammation-induced cytokines (such as TNF-α and IL-6) from the endoplasmic reticulum to the cell surface occurs through the endosomal system, and relies on a complex array of trafficking machinery to ensure the accurate docking and fusion of carrier vesicles to their designated target membranes [117]. Delivery and internalization of membrane receptors in macrophages are essential for the regulation of cell activation [118], and their trafficking also occurs through endosomes. Notably, Rab7 is fundamental for the trafficking of receptor-containing vesicles involved in the inflammatory response [119], thus supporting the notion that LRRK2 might affect the expression of membrane receptors on microglia cells.

The endosomal pathway also plays a central role during phagocytosis. Macrophages, when in contact with pathogens or dying cells, initiate a sequence of membrane fusion events that culminate with the formation of a phagocytic cup, which is followed by the formation of a nascent phagosome around the ingested material, and its consequent internalization [120]. The phagosome is then reshaped to form the phagolysosome, where pathogens are degraded to produce antigenic peptides. Multiple soluble N-ethylmaleimide-sensitive factor attachment protein receptor (SNARE) proteins are required for the formation and the maturation of phagosome. Specifically, phagocytic cup formation is determined by fusion of the plasma membrane with intracellular organelles including recycling endosomes, late endosomes, and endoplasmic reticulum, which have a large amount of membrane and contain the appropriate SNARE and Rab GTPase proteins. It has been demonstrated that macrophages, when activated, regulate the expression of SNAREs and GTPases to provide a large set of proteins for membrane fusion, important for phagocytic cup formation [115].

Although a role for LRRK2 in vesicle trafficking in microglia cells has not been described, we speculate that LRRK2 activity may be important for the release of inflammatory mediators, delivery of membrane receptors, and phagocytotic processes. In particular, LRRK2 might regulate the components of the fusion machinery, such as its partner NSF [31] or Rab GTPase proteins, which have been shown to contribute to the fusion of vesicles containing inflammatory mediators, receptors, and pathogens to be eliminated. It should be noted that several receptors regulating microglial activation, such as major histocompatibility complex (MHC) class II and CD11b, which have also been observed to be up-regulated in brains of patients with PD [51,121,122], showed increased mRNA expression under basal conditions in transgenic microglia with LRRK2 R1441G mutation, thus suggesting that LRRK2 might affect the expression of these receptors. Moreover, when these cells were activated with LPS, the levels of cytokines/chemokines, proteins, and mRNAs massively increased compared with wild-type cultures [79], thus indicating that LRRK2 might play a key role in vesicle trafficking and release in microglia cells and might be important in switching these cells toward a pro-inflammatory phenotype (Figure 1).

## Conclusions

Recent literature indicates that microglial LRRK2 plays a role in the cellular pathways induced by inflammatory stimuli. Based on its complex architecture with different functional domains, LRRK2 might be involved in more than one cellular process. Although a number of studies have analyzed the effects of LRRK2 kinase activity using different microglia-related readouts, such as morphological changes and inflammatory mediator release, evidence on how LRRK2 might affect these processes is currently lacking. Here we hypothesize that abnormal LRRK2 kinase activity, due to the presence of LRRK2 mutations, might modulate the phenotype of microglia through hyperpolymerization and hyperphosphorylation of cytoskeleton and vesicle components, thus pushing these cells toward a pro-inflammatory state. In this regard, Gillardon and colleagues observed that the LRRK2 R1441G mutation sensitizes microglia cells toward a neurotoxic state [79]. Similar to G2019S, the R1441G mutation causes an approximately 2.5-fold increase of serine 1292 autophosphorylation in cells overexpressing LRRK2-R1441G compared with wild-type LRRK2 [22], supporting the notion that the increased kinase activity of LRRK2 may push microglia toward a pro-inflammatory phenotype. One possibility is that the R1441G mutation increases kinase activity through its reduced GTP hydrolysis activity, although the cross-talk between GTPase and the kinase domain is currently unclear [12,21,123].

The identification of microglia-specific kinase substrates, GTPase downstream effectors, and interactors, will provide valuable insight into the emerging function of LRRK2 in the regulation of neuroinflammation, with the potential of uncovering new molecular mechanisms underlying the pathogenesis of PD, and consequently novel targets for drug treatments.

## Abbreviations

CNS: Central nervous system; COR: C-terminus of ROC; ERM: ezrin, radixin, moesin; GWAS: Genome-wide association study; HLA: Human leukocyte antigen; iNOS: Inducible nitric oxide synthase; IFN: Interferon; IL: Interleukin; LPS: Lipopolysaccharide; LRRK2: Leucine-rich repeat kinase 2; MHC: Major histocompatibility complex; MPTP: 1-methyl-4-phenyl-1,2,3,6-tetrahydropyridine; NFAT: nuclear factor of activated T cells; NF-κB: nuclear factor kappa B; NRON: Noncoding (RNA) repressor of nuclear factor of activated T cells; NSF: N-ethylmaleimide-sensitive factor; PD: Parkinson’s disease; ROC: Ras of complex proteins; SNARE: Soluble N-ethylmaleimide-sensitive factor attachment protein receptor; SNpc: Substantia nigra pars compacta; SV2A: Synaptic vesicle glycoprotein 2A; TNF: Tumor necrosis factor; TLR: Toll-like receptor.

## Competing interests

The authors declare that they have no competing interest.

## Authors’ contributions

IR and EG conceived the paper; IR, LB, and EG wrote the paper. All authors read and approved the final manuscript.
